# Calculating genetic risk for dysfunction in pleiotropic biological processes using whole exome sequencing data

**DOI:** 10.1186/s11689-022-09448-8

**Published:** 2022-06-24

**Authors:** Olivia J. Veatch, Diego R. Mazzotti, Robert T. Schultz, Ted Abel, Jacob J. Michaelson, Edward S. Brodkin, Birkan Tunc, Susan G. Assouline, Thomas Nickl-Jockschat, Beth A. Malow, James S. Sutcliffe, Allan I. Pack

**Affiliations:** 1 Department of Psychiatry and Behavioral Sciences, Medical Center, University of Kansas, Kansas City, KS USA; 2 Division of Medical Informatics, Department of Internal Medicine, Medical Center, University of Kansas, Kansas City, KS USA; 3 Center for Autism Research, Children’s Hospital of Philadelphia, Philadelphia, PA USA; 4 Department of Neuroscience and Pharmacology, Iowa Neuroscience Institute, University of Iowa, Iowa City, Iowa, USA; 5 Department of Psychiatry, University of Iowa, Iowa City, Iowa, USA; 6 Department of Psychiatry, Perelman School of Medicine, University of Pennsylvania, Philadelphia, PA USA; 7 Belin-Blank Center for Gifted Education and Talent Development, University of Iowa, Iowa City, Iowa, USA; 8 Division of Sleep Medicine, Department of Neurology, Vanderbilt University Medical Center, Nashville, TN USA; 9 Department of Molecular Physiology and Biophysics, Vanderbilt Genetics Institute, Vanderbilt University, Nashville, TN USA; 10 Division of Sleep Medicine, Department of Medicine, Perelman School of Medicine, University of Pennsylvania, Philadelphia, PA USA

**Keywords:** Autism spectrum disorders, Sleep duration, Pleiotropy, Exome sequencing, Genetic risk scores, Systems biology

## Abstract

**Background:**

Numerous genes are implicated in autism spectrum disorder (ASD). ASD encompasses a wide-range and severity of symptoms and co-occurring conditions; however, the details of how genetic variation contributes to phenotypic differences are unclear. This creates a challenge for translating genetic evidence into clinically useful knowledge. Sleep disturbances are particularly prevalent co-occurring conditions in ASD, and genetics may inform treatment. Identifying convergent mechanisms with evidence for dysfunction that connect ASD and sleep biology could help identify better treatments for sleep disturbances in these individuals.

**Methods:**

To identify mechanisms that influence risk for ASD and co-occurring sleep disturbances, we analyzed whole exome sequence data from individuals in the Simons Simplex Collection (*n* = 2380). We predicted protein damaging variants (PDVs) in genes currently implicated in either ASD or sleep duration in typically developing children. We predicted a network of ASD-related proteins with direct evidence for interaction with sleep duration-related proteins encoded by genes with PDVs. Overrepresentation analyses of Gene Ontology-defined biological processes were conducted on the resulting gene set. We calculated the likelihood of dysfunction in the top overrepresented biological process. We then tested if scores reflecting genetic dysfunction in the process were associated with parent-reported sleep duration.

**Results:**

There were 29 genes with PDVs in the ASD dataset where variation was reported in the literature to be associated with both ASD and sleep duration. A network of 108 proteins encoded by ASD and sleep duration candidate genes with PDVs was identified. The mechanism overrepresented in PDV-containing genes that encode proteins in the interaction network with the most evidence for dysfunction was cerebral cortex development (GO:0,021,987). Scores reflecting dysfunction in this process were associated with sleep durations; the largest effects were observed in adolescents (*p* = 4.65 × 10^–3^).

**Conclusions:**

Our bioinformatic-driven approach detected a biological process enriched for genes encoding a protein–protein interaction network linking ASD gene products with sleep duration gene products where accumulation of potentially damaging variants in individuals with ASD was associated with sleep duration as reported by the parents. Specifically, genetic dysfunction impacting development of the cerebral cortex may affect sleep by disrupting sleep homeostasis which is evidenced to be regulated by this brain region. Future functional assessments and objective measurements of sleep in adolescents with ASD could provide the basis for more informed treatment of sleep problems in these individuals.

**Supplementary Information:**

The online version contains supplementary material available at 10.1186/s11689-022-09448-8.

## Background

Autism spectrum disorder (ASD) is characterized by impairments in social interactions and social communication, as well as a pattern of restricted and repetitive interests and/or behaviors [1]. ASD etiology has contributions from common and rare variations affecting numerous genes [2, 3]. In addition, many individuals with ASD have co-occurring conditions that are important to recognize when developing treatment regimens (e.g., intellectual disability, sleep disturbances) [4].

Sleep problems, notably insomnia-related symptoms, are some of the most common issues reported in individuals with ASD [5]. Evidence indicates that sleep problems are associated with symptom severity [6, 7] and that treating sleep disturbances effectively can improve other daytime symptoms [8]. Identifying more personalized, effective treatment for sleep problems in individuals with ASD is an important area of research. By understanding the causes of sleep problems in different individuals with ASD, there is an opportunity to develop more effective approaches for treating one of the most prevalent conditions observed to co-occur with ASD.

Evidence indicates that numerous genetic loci are associated with multiple traits [9] and that, in lieu of sharing specific genes and variants, many complex diseases are connected by similar underlying molecular pathways [10]. Many of the same genes with ASD-associated variation have evidence for pleiotropic effects on regulation of sleep and circadian rhythms [11–15]. Furthermore, ASD candidate genes encode proteins that function in the same biological systems that relate to the functions of sleep to promote plasticity and connectivity during neurodevelopment [16]. For example, numerous genes cited in connection with ASD as well as those associated with variability in sleep-related traits encode proteins important to synaptic function; sleep is also evidenced to be important for synaptic plasticity [16–18]. Identifying pleiotropic mechanisms connecting ASD and insomnia-related traits may inform future work focused on establishing novel targets for sleep interventions in these patients.

We hypothesized that there are pleiotropic genetic mechanisms connecting risk for ASD with variability in sleep duration and that scores predicting dysfunction in these mechanisms would associate with insomnia-related symptoms in individuals with ASD. Importantly, large datasets are necessary to have power to detect most genetic effects influencing ASD and sleep-related information in large ASD datasets are limited to parent reports. Our previous work comparing sleep habit questionnaires to actigraphy measurements has shown that sleep duration (SD) is the most reliable insomnia-related trait that can be derived from parent report [19]. To determine if dysfunction in pleiotropic genetic mechanisms connecting ASD and sleep influence sleep duration in individuals with ASD, we first identified predicted damaging variants in candidate genes for ASD (https://gene.sfari.org/) and for childhood sleep duration [20] using WES data from the Simons Simplex Collection (SSC; [21]). We then predicted a protein–protein interaction (PPI) network connecting ASD gene products with SD gene products and identified Gene Ontology biological processes that were enriched for genes encoding proteins in the PPI network. We developed an equation to calculate the likelihood of genetic dysfunction in the significantly enriched biological processes and tested for associations with parent-reported sleep durations from the SSC. We found that incorporating evidence from predicted protein damaging variants located in ASD and SD candidate genes with evidence for protein–protein interactions revealed specific mechanisms associated with sleep duration in individuals with ASD. Notably, the approaches described and reported scores can be adapted to analyze WES data for different co-occurring conditions in ASD and other intellectual and developmental disabilities.

## Methods

### Prediction of protein damaging variants using whole-exome sequence data

To detect genes with potential pleiotropic effects underlying expression of ASD with co-occurring sleep disturbances, we first identified currently implicated ASD and sleep duration (SD) candidate genes with predicted damaging variants (PDVs) in the Simons Simplex Collection (SSC) dataset provided by the Simons Foundation (SSC Whole-exome Sequences 3 (NextCODE)). The SSC represents the largest collection of simplex ASD families, with one affected child (i.e., proband) and at least one unaffected sibling. Sex discrepancies were identified using both the ratio of heterozygous SNVs on the X chromosome compared to autosomes and coverage of the Y chromosome gene, *SRY*. There were 12 individuals with unclear gender assignments where genetic sex could not be determined or who had 47,XYY or 47,XXX; these individuals were excluded from analyses. The final analysis dataset included 2380 individuals; this was 86% male and 79% reported white (Table S1).

ASD candidate genes were those included in the most recent update (2021 Q2) of the Simons Foundation Autism Research Initiative human gene database (SFARI, https://gene.sfari.org/database/human-gene [22, 23]). SD candidate genes were derived from genome-wide studies of an independent population of children with no evidence of ASD. Specifically, genes were selected based on gene-based test results—available via the Sleep Disorder Knowledge Portal (https://sleep.hugeamp.org/) that were conducted using summary statistics from a genome-wide association study (GWAS) of sleep duration in more than 11,000 assumed typically developing children, ages 2–13 years old, with European ancestry [20].

SNVs and indels (< 200 bp) were selected from previously processed WES data. Genomic locations were based on Human Genome Build GRCh37/hg19. SNVs and indels were previously called across all 22 autosomes and both sex chromosomes using the Genome Analysis Toolkit (GATK; [24]) and FreeBayes [25] software. Detailed information can be found in Iossifov et al. 2014 [26]. Quality control (QC) thresholds were set at a depth ≥ 8 reads, genotype quality of ≥ 20, and exclusion of variants not passing GT filter criteria. Validated CNVs previously reported in Sanders et al. [27] and Krumm et al. [28] were also included. Bedtools [29] was used to identify regions of overlap between CNVs reported across these previously published studies.

To assess the likelihood that a variant was damaging to the protein-coding gene products, we ran Variant Effect Predictor (VEP, [30]) and prioritized SNVs and indels with consequences that were highly likely to be damaging (i.e., splice site alterations, gains or losses of stop codons, loss of start codons, or frameshifts). Nine additional prediction algorithms were run using filter-based annotation from ANNOtate VARiation (ANNOVAR) software [31]. Algorithms included in ANNOVAR that were used to predict damaging variants were (1) sorts intolerant from tolerant (SIFT) based on sequence homology and the physical properties of amino acids[32]), (2) polymorphism phenotyping v2 (Polyphen-2) HVAR based on structural and comparative evolutionary considerations [33], (3) mutation taster based on protein features, genotype frequencies, evolutionary conservation, splice-site changes, and mRNA stability [34], (4) mutation assessor based on predicted specificity residues [35], (5) likelihood ratio test (LRT) based on evolutionary conservation [36], (6) functional analysis through hidden Markov Models (FATHMM-MKL) based on experimentally validated functional elements within the human genome and sequence homology [37], (7) protein variation effect analyzer (PROVEAN) based on sequence homology [38], (8) MetaLR which is a meta predictor trained on an ensemble of approaches [39], and (9) Mendelian clinically applicable pathogenicity (M-CAP) which is also a meta predictor trained on an ensemble of approaches [40]. For additional summary-level details on these as well as other available in silico predictors please refer to Dong et al. [39] and Jagadeesh et al. [40]. The following equation was used to calculate the possibility a variant was damaging:

$${\varvec{P}}{\varvec{D}}{\varvec{V}}={\varvec{F}}{\varvec{D}}\times {\varvec{Z}}$$

where ${\varvec{P}}{\varvec{D}}{\varvec{V}}=$ predicted damaging variant;${\varvec{F}}{\varvec{D}}=\left(\left({\varvec{D}}-{\varvec{B}}\right)+1\right))/\left({\varvec{N}}+1\right)$ where ${\varvec{F}}{\varvec{D}}=$ frequency of damaging predictions, ${\varvec{D}}=$ algorithms calling the variant damaging, ${\varvec{B}}=$ algorithms calling the variant benign, ${\varvec{N}}=$ total number of algorithms providing a prediction, and $1=$ constant accounting for variants being preselected using VEP, and ${\varvec{Z}}=$ zygosity (heterozygous = 1, homozygous = 2).${\varvec{F}}{\varvec{D}}$ scores ranged from − 0.8 − 1.0 with a negative score indicating the variant was more often predicted benign. Considering the focus was on deleterious variants, negative ${\varvec{F}}{\varvec{D}}$ scores were assumed to be zero. Males with heterozygous X chromosome variants in pseudoautosomal regions (PAR) were weighted as autosomal variants and those located outside of PAR1 and PAR2 were considered homozygous. CNVs encompassing portions of the coding (i.e., exonic, splice-site) and proximal promoter (i.e., 5′-UTR) regions of genes were given weights equal to SNVs and indels with the strongest likelihood of being damaging and assumed heterozygous. To distinguish de novo from inherited variants, we used previously published data [27].

### Prediction of pleiotropic biological processes connecting ASD and SD

We then predicted protein–protein interactions between PDV-containing ASD candidate proteins and PDV-containing SD candidate proteins using STRINGdb v11 [41]. Overrepresentation analyses were conducted comparing genes encoding ASD-related proteins with evidence for direct interactions with proteins encoded by SD genes in Gene Ontology (GO) biological processes [42, 43] to all human protein coding genes included in Ensembl release 99. The weight01 algorithm from the TopGO package version 2.38.1 was used to perform overrepresentation analyses [44, 45]. The significance was determined using Fisher’s exact test; the threshold was set at a false discovery rate-adjusted *α* < 0.05. GO term definitions were based on AmiGO 2 version 2.5.13, GO version 2020–11-90; doi:10.5281/zenodo.4281619 [46]. All genes with PDVs assigned to significantly overrepresented processes were then identified. The overlap among PDV-containing genes assigned to different significantly overrepresented biological processes was visualized using the UpSetR package v1.4.0.

Overrepresentation analyses were also conducted to determine if the significance of results for GO biological processes that were enriched for genes encoding proteins in the ASD and SD PPI network were different when compared to results obtained with the entire candidate gene sets.

### Calculation of overall biological process dysfunction

We developed the following equation to calculate scores reflecting the likelihood of dysfunction in overall biological processes:

$${\varvec{D}}{\varvec{B}}{{\varvec{P}}}_{{\varvec{X}}}=\sum \left({\varvec{P}}{\varvec{D}}{{\varvec{V}}}_{{\varvec{v}}{\varvec{n}}}^{{\varvec{G}}{\varvec{e}}{\varvec{n}}{\varvec{e}}{\varvec{A}}}\times {\varvec{E}}{\varvec{B}}{{\varvec{P}}}_{{\varvec{X}}}^{{\varvec{G}}{\varvec{e}}{\varvec{n}}{\varvec{e}}{\varvec{A}}}\right)/{\varvec{n}}{\varvec{G}}{\varvec{e}}{\varvec{n}}{\varvec{e}}{\varvec{s}}{\varvec{B}}{\varvec{P}}{\varvec{x}}$$

where ${\varvec{D}}{\varvec{B}}{{\varvec{P}}}_{{\varvec{X}}}$= dysfunction of a given biological process—represented in the equation as “*BP*_*x*_”; (${\varvec{P}}{\varvec{D}}{{\varvec{V}}}_{{\varvec{v}}{\varvec{n}}}^{{\varvec{G}}{\varvec{e}}{\varvec{n}}{\varvec{e}}{\varvec{A}}}$) = the sum of the PDV scores for each distinct variant, represented in the equation as “*vn*”, identified in a given gene, represented in the equation as “*GeneA”*; (${\varvec{E}}{\varvec{B}}{{\varvec{P}}}_{{\varvec{X}}}^{{\varvec{G}}{\varvec{e}}{\varvec{n}}{\varvec{e}}{\varvec{A}}} )$ is the sum of the frequencies of GO evidence codes for each gene—where frequencies are based on evidence codes used for all genes assigned to the process—supporting assignment to the process, plus the number of “child term” biological processes that were in the same branch of the GO hierarchy as the significant “parent term”, divided by the total number of child terms included in the GO hierarchy for the process; and (${\varvec{n}}{\varvec{G}}{\varvec{e}}{\varvec{n}}{\varvec{e}}{\varvec{s}}{\varvec{B}}{\varvec{P}}{\varvec{x}}$) = the total number of genes assigned to the biological process.

To assess score distributions and normality assumptions, violin plots for DBP scores were plotted and Shapiro–Wilk’s tests conducted. DBP score correlations were calculated using pairwise Spearman’s rank correlations (*ρ*), and distributions by process were visualized with violin plots. In addition, the proportion of individuals with any evidence of dysfunction (DBP > 0) compared to no evidence of dysfunction (DBP = 0) was calculated; the process with the highest evidence of dysfunction was selected to test for associations with sleep-related traits—described below. The predicted ASD protein-SD protein interaction network of genes assigned to the selected overrepresented process was visualized using Cytoscape v3.8.2 [47].

### Approach validation using random genes matched for ASD or SD gene features

To help validate that the approach was not simply identifying processes overrepresented for genes that more often generate false positive indel calls in WES due to molecular features, two random sets of genes were evaluated. Random gene set one was matched to ASD candidate genes, and random gene set two was matched to SD candidate genes. To match features across these gene sets, the mean guanine-cytosine (GC) content, transcript size, and the number of spliceoforms for the ASD and SD gene sets were calculated. Two random sets were then selected from all human protein coding genes included in Ensembl release 99, excluding the ASD and SD candidate genes, based on similar mean GC content, transcript size, and number of spliceoforms to their candidate gene set counterpart using the fuzzyjoin package v0.1.6 in R. Each random, feature-matched gene set reflected the same number of distinct genes that were included in the ASD (*n* = 1014) and SD candidate genes (*n* = 837). Molecular features of the final random sets were compared to the features of the candidate gene sets using *t* tests.

We then ran our analysis pipeline described above on randomly selected genes as follows: (1) identified PDVs in random gene set matched to ASD and compared the total number of genes with PDVs in the random ASD-matched set to the ASD set as well as the (2) identified PDVs in random set matched to SD and compared the total number of genes with PDVs in the random SD-matched set to the SD set, (3) predicted PPI between ASD-matched random genes and SD-matched random genes, and (4) ran overrepresentation analyses comparing random genes with PDVs in PPI to the entire gene universe.

### Tests for associations between biological process genetic risk scores and sleep duration

Linear least squares regression was used to test if the DBP score with the most evidence for dysfunction was associated with parent-reported sleep duration available in proband medical history intakes provided by the SSC for a portion of the dataset (*n* = 2288 with WES and sleep data). For information on medical history data collection see Fischbach and Lord, 2010 [21]. Sleep duration was determined using current answers to the question “On average, how many hours/night [does your child sleep]?” as described in our previous study [6]. Regression was also used to test for associations between DBP scores and having a sleep duration determined to be “extremely short” (i.e., ≤ 420 min, or the lower 5th percentile of the distribution; *n* = 144), “non-extreme” (i.e., > 420 but < 660 min, or between the 5th and 95th percentiles; *n* = 1900), or “extremely long” (i.e., ≥ 660 min or the upper 95th percentile; *n* = 244) also described in our previous study [6]. All tests were conducted while adjusting for age at ascertainment, genetically determined sex, reported race and intellectual quotient (IQ) scores. As we had previously observed that sleep duration was associated with social/communication impairment and restricted repetitive behaviors (RRB) in this dataset [6], we evaluated additional models that included social/communication impairment and RRB reported on the autism diagnostic interview-revised and measured on the autism diagnostic observation schedule. We based significance on a *p* value < 0.05. To determine if age or sex modified associations, we performed interaction tests including a product term in the model with main effects.

## Results

### Dysfunctional biological processes influencing ASD and Sleep

At the time of these analyses, there were 1014 different protein coding ASD candidate genes included in the SFARI database. There were 612 ASD candidate genes with VEP high consequence variants that were more often predicted by in silico algorithms to be damaging than benign (Table S2). Every proband had a PDV in a currently implicated ASD gene with PDVs in 16 different ASD genes, on average (x̅ = 15.76 ± 3.94).

There were 837 SD candidate genes where gene-based tests using summary statistics from a published GWAS of sleep duration in children were significant at *p* < 0.05 [20]. Of these, 505 currently implicated SD genes had VEP high consequence variants predicted damaging more often than benign in the ASD dataset. All but one proband had a PDV in a currently implicated SD gene. On average, each individual had a PDV in seven different SD genes (x̅ = 7.24 ± 2.70). Across all genes with PDVs in the SSC dataset, 29 were separately implicated in both ASD (from SFARI gene) and SD in children (from sleep duration GWAS) (Table S2).

There were 819 PDV-containing candidate genes for ASD and/or SD that encoded proteins with direct evidence for interaction (Table S2), and nine biological processes overrepresented in these genes (Table 1). Significant terms were “*GO:0,035,176: social behavior*”, “*GO:2,000,463: positive regulation of excitatory postsynaptic potential*”, “*GO:0,086,010: membrane depolarization during action potential*”, “*GO:0,051,968: positive regulation of synaptic transmission*”, “*GO:0,019,228: neuronal action potential*”, “*GO:0,021,987: cerebral cortex development*”, “*GO:2,000,310: regulation of NMDA receptor activity*”, “*GO:0,007,158: neuron cell–cell adhesion*”, and *“GO:0,034,765: regulation of ion membrane transport”*. Most PDV-containing genes were assigned to only one process (x̅ = 1.24 ± 0.62; Figure S1), indicating these were genetically distinct. Results evaluating biological processes that were overrepresented for the entire set of ASD and SD candidate genes showed that “*GO:0,019,228: neuronal action potential*”, “*GO:0,021,987: cerebral cortex development*”, “*GO:2,000,310: regulation of NMDA receptor activity*”, and *“GO:0,034,765: regulation of ion membrane transport”* no longer met the FDR-adjusted threshold for significant enrichment (Table 1).

**Table 1:** Processes overrepresented for proteins in ASD/SD protein–protein interaction network

GO.ID	Term	Anno	Sig	Exp	FE	p-value	FDR	FDR _All ASD&SD Genes_
GO:0,035,176	social behavior	51	16	2.59	5.79	1.94 × 10^–8^	3.11 × 10^–4^	6.23 × 10^–4^
GO:2,000,463	positive regulation of excitatory postsynaptic potential	28	12	1.42	7.75	5.24 × 10^–8^	4.19 × 10^–4^	1.64 × 10^–3^
GO:0,086,010	membrane depolarization during action potential	36	11	1.83	6.01	1.09 × 10^–6^	5.80 × 10^–3^	1.15 × 10^–2^
GO:0,051,968	positive regulation of synaptic transmission	32	10	1.62	6.17	2.50 × 10^–6^	1.00 × 10^–2^	1.69 × 10^–2^
GO:0,019,228	neuronal action potential	34	10	1.73	5.78	4.64 × 10^–6^	1.37 × 10^–2^	1.11 × 10^–1^
GO:0,021,987	cerebral cortex development	117	20	5.94	3.37	5.12 × 10^–6^	1.37 × 10^–2^	1.20 × 10^–1^
GO:2,000,310	regulation of NMDA receptor activity	36	10	1.83	5.46	8.20 × 10^–6^	1.87 × 10^–2^	1.00 × 10^–0^
GO:0,007,158	neuron cell–cell adhesion	17	7	0.86	8.14	1.05 × 10^–5^	2.11 × 10^–2^	1.15 × 10^–2^
GO:0,034,765	regulation of ion membrane transport	473	50	24.01	2.08	1.86 × 10^–5^	3.31 × 10^–2^	8.96 × 10^–2^

Shown are significant results, based on a false discovery rate (FDR)-corrected threshold, from conditional gene set overrepresentation analyses of ASD and sleep duration (SD) candidate genes with predicted damaging variants in the Simons Simplex Collection that encoded proteins with direct evidence for interaction. Abbreviations: *GO* Gene Ontology, *Anno* Genes annotated in process, *Sig* PDV-containing gene assigned to process, *Exp* Number of genes expected to be assigned to process by chance, *FE* Fold enrichment (Sig/Exp). *P*-values represent the probability that PDV-containing genes would be assigned to the biological process by chance. Also included is the FDR-adjusted *p*-value for analyses run using all ASD and SD candidate genes regardless of evidence for interactions

**Fig. 1:**
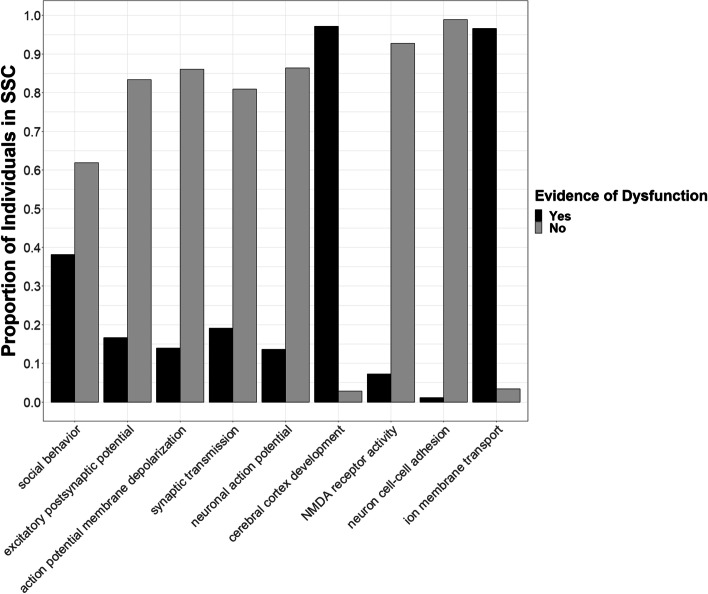
Evidence of any dysfunction in pleiotropy network biological processes in individuals with ASD. Plotted are the proportion of individuals with autism spectrum disorder (ASD) with evidence of dysfunction (DBP > 0) versus no evidence of dysfunction (DBP = 0) in biological processes with overrepresentation of ASD and/or sleep duration (SD) genes in the ASD-SD protein–protein interaction network

**Fig. 2:**
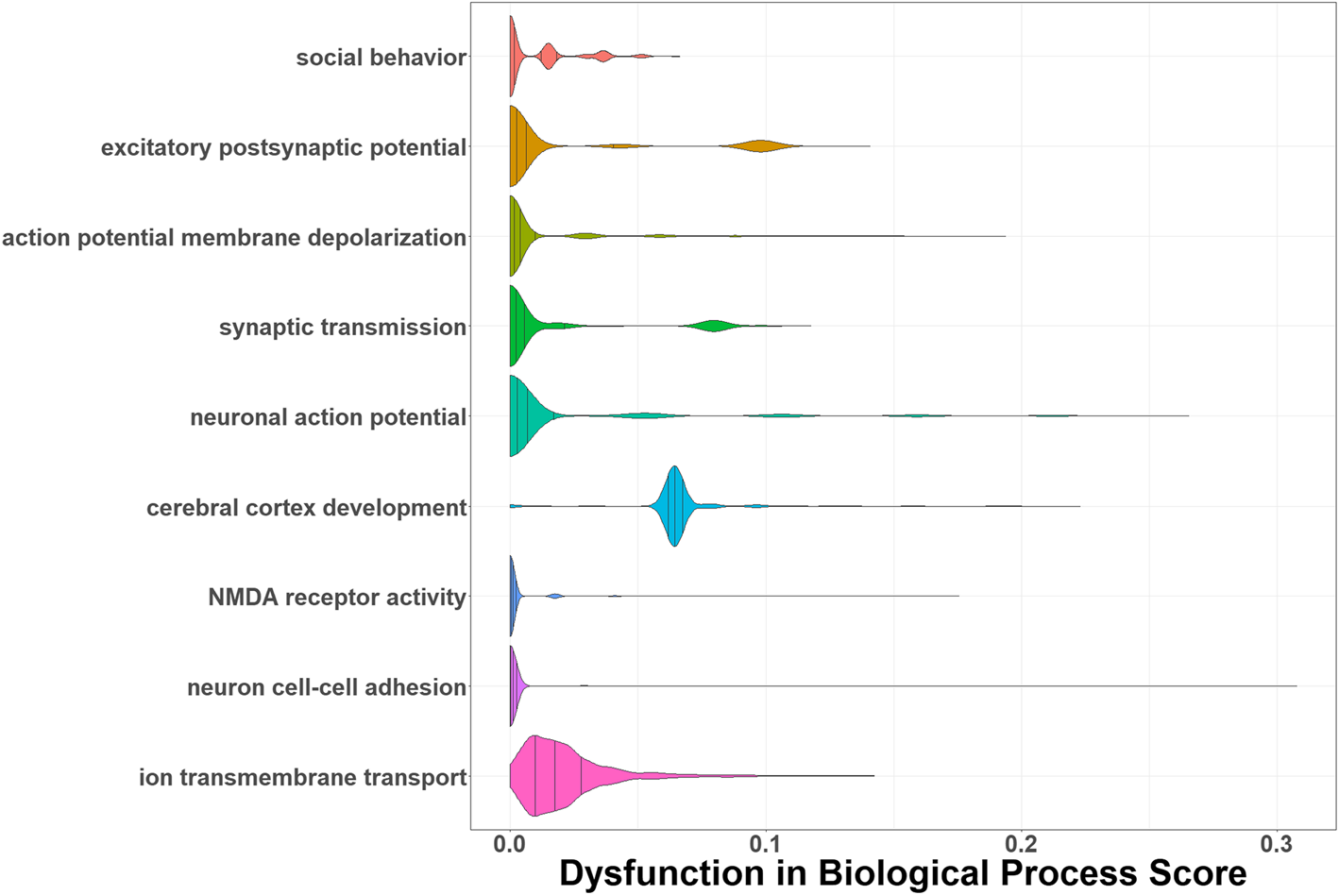
Genetic risk scores reflecting the level of dysfunction in pleiotropy network biological processes in individuals with ASD. Shown are the distributions of raw scores, across individuals with autism spectrum disorder (ASD), for each process with significantly more PDV-containing genes encoding proteins in the ASD-sleep duration protein–protein interaction network. No scores were normally distributed (*p* ≤ 8.46 × 10^–48^)

**Fig. 3:**
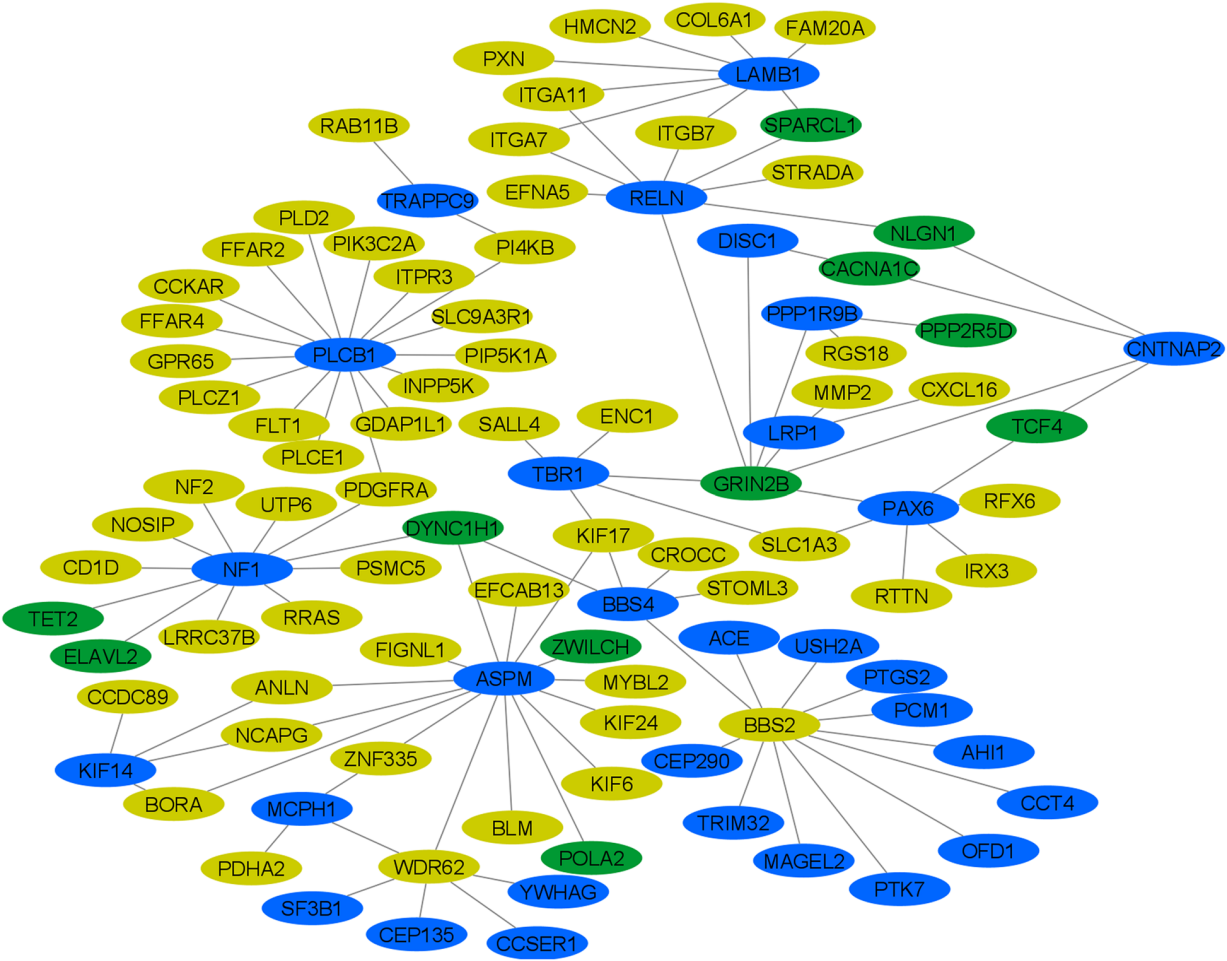
Network of proteins encoded by autism spectrum disorder (ASD) and sleep duration (SD) genes implicated in cerebral cortex development. Shown is the protein–protein interaction network predicted for the products of genes with predicted damaging variants identified in individuals with ASD that are assigned to the Gene Ontology biological process of “GO:0,021,987: cerebral cortex development”. Proteins are colored according to the associated condition as follows: blue = ASD-related protein, yellow = SD-related protein, green = both ASD, and SD-related protein

The majority of the dataset (*n* = 2368 or 99.5%) had evidence for dysfunction in at least one process (Fig. 1). On average, each individual had a DBP score greater than zero in three processes (x̅ = 3.05, sd = 1.41). All DBP scores were significantly different from a normal distribution (*p* ≤ 8.46 × 10^–48^, Fig. 2). The process with the most evidence of genetic dysfunction in the ASD dataset was “cerebral cortex development” (Figs. 1 and 2). Scores reflecting dysfunction in this process were correlated with other DBP scores for *membrane depolarization during action potential* (*ρ* = 0.19, *p* = 1.0 × 10^–9^), *positive regulation of synaptic transmission* (*ρ* = 0.04, *p* = 3.01 × 10^–2^), *neuronal action potential* (*ρ* = 0.21, *p* = 1.0 × 10^–9^), *regulation of NMDA receptor activity* (*ρ* = 0.28, *p* = 1.0 × 10^–9^), and *regulation of ion membrane transport* (*ρ* = 0.17, *p* = 4.44 × 10^–16^; Figure S2). There were 108 genes with PDVs in the ASD/SD pleiotropy network predicted to influence “cerebral cortex development” in humans based on evidence from Gene Ontology (Fig. 3).

### Comparisons with randomly selected, feature-matched gene sets

There were fewer random genes with PDVs (*n* = 605 random versus 612 ASD) when comparing the gene set matched for features in the ASD candidate gene set and similarly for random genes matched to the SD candidate gene set (*n* = 472 random versus *n* = 505 SD). On average, individuals had more ASD (*t* = 22.88 [df = 3317], *p* < 2.2 × 10^–16^) and fewer SD (*t* =  − 5.26 [df = 4649], *p* = 1.49 × 10^–7^) candidate genes with PDVs compared to random genes with PDVs (Table S3). A PPI network was predicted between random genes feature-matched to ASD gene and random genes feature-match to SD genes; however, there were no biological processes overrepresented for random genes with PDVs encoding proteins in this predicted PPI network that met the FDR-adjusted significance threshold (Table S3). Since no biological processes overrepresented for the PPI network of random genes were identified, no subsequent scores were calculated, and the remaining steps in the analysis pipeline could not be completed.

### DBP scores for cerebral cortex development associate with sleep duration

**Fig. 4:**
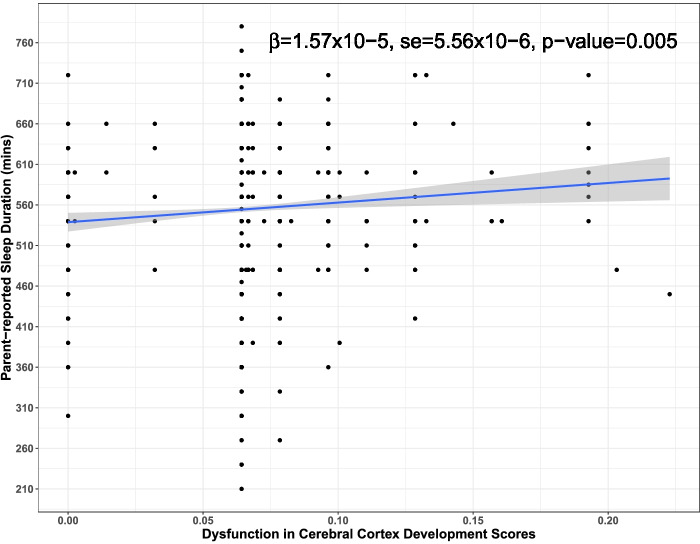
Association between dysfunctional cerebral cortex development scores and sleep duration. Plotted is the linear prediction for the relationship between dysfunctional biological process (DBP) scores for cerebral cortex development (GO:0,021,987) and reported sleep duration in minutes. 95% confidence intervals around fitted lines are indicated in gray; Beta coefficients (*β*) and the corresponding *p* value are provided

Increased evidence for dysfunction in the Gene Ontology-defined process of “cerebral cortex development” was associated with a small effect (*β* = 0.000016 [0.0000006], *p* = 4.87 × 10^–3^) on longer parent-reported sleep duration (Fig. 4; Table S4a). In particular, genetic scores reflecting evidence for dysfunction in this biological process had the strongest effects on having a sleep duration that was extremely long, or ≥ 11 h, based on the overall distribution of sleep durations reported in this dataset (*β* = 0.0063 [0.0019], *p* = 1.2 × 10^–3^; Table S4b). These effects remained significant when adjusting for ASD core symptom severity in regression models (Table S4a-b). Interaction tests indicated that age had a near significant effect on the association between DBP scores for “cerebral cortex development” and having a sleep duration that was extremely long (*β* = 0.0001 [0.00005], *p* = 8.43 × 10^–2^; Table S4c). The largest effect of DBP scores on sleep duration was observed in individuals who were teenagers of secondary-school age (13–18 years old) when compared to either young preschool age (4–5 years old) or primary school-age (6–12 years old) children (*β* = 0.00005 [0.00002], *p* = 4.65 × 10^–3^; Table 2, Table S4d).

**Table 2:** Associations between genetic evidence of dysfunction in cerebral cortex development and sleep duration in different age groups

Phenotype Variable	β†	SE	t-statistic	*p*-value
**Preschool Age Children (*n* = 396)**				
Sleep Duration (240–780 min)	2.32 × 10^–5^	1.15 × 10^–5^	2.01	4.47 × 10^–2^
Full Scale IQ (range = 19–150)	-1.13 × 10^–5^	3.36 × 10^–5^	-0.34	7.36 × 10^–1^
Age (range = 4–5 years)	-1.15 × 10^–4^	1.61 × 10^–4^	-0.71	4.76 × 10^–1^
Male Sex (*n* = 339)	-4.34 × 10^–4^	2.47 × 10^–3^	-0.18	8.60 × 10^–1^
**Primary School-Age Children (*n* = 1,467)**				
Sleep Duration (240–720 min)	1.88 × 10^–6^	6.71 × 10^–6^	0.28	7.79 × 10^–1^
Full Scale IQ (range = 10–165)	6.46 × 10^–6^	1.70 × 10^–5^	0.38	7.04 × 10^–1^
Age (range = 6–12 years)	-4.02 × 10^–5^	2.02 × 10^–5^	-1.99	4.72 × 10^–2^
Male Sex (*n* = 1,273)	-5.30 × 10^–4^	1.34 × 10^–3^	-0.40	6.93 × 10^–1^
**Secondary School-Age Teenagers (*n* = 425)**				
Sleep Duration (210–720 min)	4.71 × 10^–5^	1.66 × 10^–5^	2.85	4.65 × 10^–3^
Full Scale IQ (range = 7–161)	-5.13 × 10^–5^	3.47 × 10^–5^	-1.48	1.39 × 10^–1^
Age (range = 13–18 years old)	1.28 × 10^–4^	5.83 × 10^–5^	2.19	2.90 × 10^–2^
Male Sex (*n* = 362)	-5.59 × 10^–3^	3.01 × 10^–3^	-1.86	6.40 × 10^–2^

Shown are results from tests for a relationship between dysfunctional biological process (DBP) scores for cerebral cortex development (GO:0,021,987) and parent-reports of sleep durations in *n* = 2,288 individuals diagnosed with ASD. Tests were conducted within different age groups based on school age. Models adjusted for intellectual quotients, reported age at ascertainment, genetically-determined sex and reported race. Provided are ranges for quantitative measures, or the number of individuals in each category for categorical variables. †For each one unit increase in the DBP score, the β-coefficient reflects the amount each measure either decreases or increases. P-values represent the probability that there is no effect

## Discussion

Our approach to analysis of WES data incorporated evidence from typical forms of variation (i.e., de novo, inherited, rare, common, SNVs, indels, and CNVs) into cumulative risk scores reflecting the likelihood for dysfunction in entire systems evidenced to have pleiotropic effects on expression of ASD and sleep duration. Calculating these scores may have helped detect the mechanisms associated with variable expressivity of sleep duration in individuals with ASD. This has implications for future work aimed at identifying more effective approaches for treating sleep problems in these patients. In general, polygenic risk scores have been shown to be useful to exploring whether genetic susceptibility underlies variable expressivity of symptoms [48] and may hold promise for informing precision medicine by offering the opportunity to predict risk and aid in early detection and intervention for many complex conditions [49]. Sleep disruption is a complex condition that may be a modifiable risk factor for increased symptom severity in individuals with ASD [8]. It is possible that estimating genetic risk contributing to expression of sleep problems could help identify those individuals with ASD who would benefit from more comprehensive sleep evaluations.

### Dysfunction in cerebral cortex development is associated with sleep duration in ASD

We observed that the accumulation of predicted damaging variants in genes encoding proteins that comprise a network connecting ASD and sleep biology point to cerebral cortex development. In general, this biological process appeared to be genetically distinct from other overrepresented processes. The majority of genes assigned to cerebral cortex development (*n* = 101/117) based on evidence included in Gene Ontology were not assigned to other processes. DBP scores calculated in the ASD dataset for cerebral cortex development were however moderately correlated with DBP scores reflecting increased dysfunction in generation of a neuronal action potential (GO:0,019,228) and regulation of *N*-methyl-d-aspartate selective glutamate receptor activity (GO: 2,000,310). DBP scores for these two other processes were strongly correlated with each other (*ρ* = 0.55, *p* = 1.00 × 10^–9^), but did not have any genes that were assigned to them both (Figure S1). Notably, glutamate serves as the major excitatory neurotransmitter in the brain and glutamate abnormalities have been observed in many individuals with ASD [50]. In addition, cortical excitatory neurotransmissions are wake-promoting [51]. It is possible that dysfunction in cerebral cortex development impacts these wake-promoting signals and results in longer periods of sleep. It is evidenced that the cerebral cortex regulates sleep through the homeostatic process. Cortical oscillations are observed to coordinate with sleep homeostasis which influences both the duration and intensity of sleep [52]. Namely, sleep homeostasis refers to the build-up in sleep pressure that accumulates during periods of wakefulness. Following prolonged periods of sleep deprivation, the majority of subsequent sleep during the recovery period is spent in slow-wave sleep which is the deepest phase of non-rapid eye movement (NREM) sleep. Conversely, prolonged periods of sleep are usually followed by a reduction in sleep propensity [53]. Studies in mice observed that cortical structures actively contribute to sleep homeostasis and the global control of vigilance states [54]. In addition, a subset of GABAergic cortical interneurons that produce nitric oxide synthase are sleep promoting and primarily active during NREM sleep [51]. Mice lacking neuronal nitric oxide synthase have less NREM sleep and a blunted homeostatic response to sleep deprivation [55]. Objective measures of human sleep following periods of deprivation have shown that the build-up of homeostatic sleep pressure is slower in mature typically developing adolescents compared with prepubertal or early pubertal children [56]. This indicates that the delay in sleep phase which is well-described in typically developing mature adolescents relates to the homeostatic regulation of sleep [56]. As such, it is possible that the homeostatic regulation of sleep in some individuals with ASD may be disturbed and that this potentially relates to predicted genetic dysfunction in cerebral cortex development.

### Genetic effects on sleep are stronger in adolescents with ASD

Notably, the most significant relationship between genetic dysfunction in cerebral cortex development and long sleep durations were observed in teenagers with ASD (ages 13–18 years old). Previous findings in ASD indicating shorter parent-reported sleep durations related to more severe core symptoms and expression of other co-occurring conditions (e.g., attention deficit, depression, obsessive compulsive disorder) were primarily driven by children who were ages 4–12 years old [6]. It is possible that we did not detect a strong relationship between having more genetic dysfunction and sleep duration in younger children because there are other non-genetic risk factors (e.g., behavioral, environmental) influencing sleep in these children. Considering the majority of the ASD dataset evaluated in this study were children within this age range (*n* = 1863, 81%), it is likely there is more heterogeneity complicating detection of shared genetic risk factors influencing expression of sleep duration variability. It is also possible that variation in genes influencing sleep duration in typically developing children are distinct from those influencing sleep durations in children with ASD.

It is also important to consider that defining sleep-related traits, particularly insomnia, is difficult where sleep-related data are being derived from potentially biased parent report. By evaluating the parent-reported sleep trait that we previously observed to be the most accurate insomnia-related trait that could be obtained from parent report [19], it is assumed that potential biases related to these third party subjective reports of sleep are minimized. This does not account for the possibility that adolescents with longer reported sleep durations also have issues related to sleep-onset delay or wake-after sleep onset which could impact sleep duration and be missed by parents and caregivers [19]. To date, very few studies have focused on understanding sleep behaviors in adolescents and adults with ASD making it difficult to draw solid conclusions regarding the results of our study. There is a profound need for more studies evaluating objective sleep data in large ASD datasets. However, the few studies that have evaluated objective-measures of sleep in adolescents and adults with ASD found that—while total sleep times were not different—older individuals with ASD spent more time in bed, had longer sleep onset latency, spent more time awake after sleep onset and had reduced sleep efficiency when compared to typically developing peers [57, 58]. This could explain the somewhat surprising observation that, primarily in adolescents with ASD, increased evidence of genetic dysfunction in cerebral cortex development was associated with longer parent-reported sleep durations when the expectation was that genetic dysfunction in sleep duration-related mechanisms would instead result in shorter sleep. As such, an extremely long parent-reported “sleep duration” could be a proxy for longer sleep onset latency and reduced sleep efficiency. Focusing on obtaining objective measures of insomnia-related traits and understanding the causes and consequences of sleep disturbances in large datasets of adolescents and adults with ASD is an important area of future work.

Furthermore, similar to the sleep durations reported in the ASD dataset, the childhood sleep duration candidate genes selected in this study were derived from results of a GWAS evaluating parent-reports of how many hours their child sleeps during the day including naps [20]. Therefore, these GWAS results may also be influenced by potentially unreliable third-party reporting of sleep traits. It is also possible that the childhood sleep duration candidate genes identified using gene-based tests are not the effector genes being tagged by GWAS hits [59]. While we focused on sleep duration genes because sleep duration was considered the most reliable phenotypic trait we could evaluate with subjective reports, it is important to confirm implicated genes using objective measures of sleep and model systems and to experimentally determine the direction of associated genetic effects. It may also be necessary to evaluate effects of predicted damaging variants in genes that influence the timing of sleep as opposed to the duration.

Another possibility is that these results reflect evidence of other co-occurring conditions that are important to treat. For example, previous studies of common genetic variants in typically developing adults detected a correlation between increased schizophrenia risk and longer sleep durations [60]. Schizophrenia often co-occurs with autism [61], and there may be shared molecular underpinnings contributing to sleep problems across these two neurodevelopmental conditions. In typically developing adults, both short and long sleep have also been observed to relate to increased risk for other mental health issues, like depression [62]. However, studies in typically developing adolescents have not observed these same relationships between long sleep and mental health problems [63]. Ultimately, objective measurements and accurate phenotyping are key to detecting true effects in computational analyses and should be a focus of future studies.

### Limitations

While limitations have been noted throughout, additional issues include the lack of independent validation of variants identified via WES to confirm that these were accurately called. This may be especially important for indels which have been observed to require additional QC to more accurately identify these via WES technologies [64]. The observation that a similar number of PDVs were identified in random gene sets compared to candidate genes SD suggests the possible presence of false positive variant calls in this WES dataset. However, the calculation of cumulative risk and the systems-based approach used in the current study should help account for potential false positive variant calls as it is assumed unlikely for many of these to congregate in the same molecular mechanism. Notably, although a PPI network was predicted we did not identify any biological processes that were overrepresented for randomly selected genes with PDVs in this “random” PPI network. This indicates that taking a systems-based approach may help account for potential false positive variant calls that are often reported in WES data.

Ultimately, the determination that a genetic variant was likely to damage to a protein product was based on in silico predictions and will require additional follow-up studies to confirm any effect on the protein function. It is unclear what the optimal approach is for in silico prediction of the likelihood a genetic variant is damaging to the encoded protein product [65–67]. As demonstrated in Supplemental Table 2, predictions from available tools vary widely when applied to the same variant as they employ different algorithms and use different training data to determine the accuracy of predictions [68]. As such, it is highly advisable to combine predictions from multiple tools to assess the overall likelihood a variant is damaging [69]. Approximately half of the variants (50.12%) that were predicted to have a negative consequence on the encoded protein based on genetic location (i.e., the VEP prediction) were not given predictions by any other algorithm. This is likely because the variant has not yet been observed in the populations that are used for training prediction algorithms. As such, it is difficult to determine the likelihood that an extremely rare variant is damaging without conducting functional follow-up studies. We also observed that ~ 19% of the SNVs and indels that were in a genetic region that was likely to be damaging were more often predicted to be benign by algorithms that incorporated information in addition to location (e.g., the frequency of the variant in populations with no evidence of disease, the level of conservation of the genetic region across species). Fortunately, as the field of in silico variant prediction continues to develop novel methods, focused on advances like mapping variants to three-dimensional protein structures [70], predictions should become more accurate and variant prioritization more efficient. Additional variant validation and functional evaluation are outside of the scope of these studies and are an important area for future work.

Another limitation relates to the possibility that predicted protein–protein interactions provided in the STRINGdb that we used to determine the pleiotropy network are not accurate. While we only selected interactions predicted at medium confidence or better, it may be necessary to select more stringent criteria for these predictions in the future. Furthermore, the GO-defined biological processes are not innately independent, and genes are assigned to multiple processes. As such, to calculate our scores for dysfunction in overall processes, genes were weighted to account for the level of evidence supporting assignment of the gene to the biological process of interest using GO evidence codes (http://www.geneontology.org/page/guide-go-evidence-codes). This could introduce bias as it is unclear what should be considered the most reliable sources of evidence supporting assignment of genes to GO terms. While experimental evidence would be preferred, it is potentially skewed, as this code will likely be assigned more often to genes that were directly evaluated for a role in the process of interest. The majority of genes are assigned to terms based on computational predictions which are considered reliable in the absence of experimental data [71]. As assignments of genes to processes are updated regularly, we have noted versions and updates of the GO used in these analyses.

Finally, while the primary goal was to determine if current evidence could be translated into clinically useful information, it is possible that by focusing on already implicated genes we did not consider all evidence for process dysfunction. Future work aimed at understanding genetic contributions to overall process dysfunction, regardless of the underlying evidence of risk for ASD may help detect more robust differences in ASD-related symptoms.

## Conclusions

Our approach identified subsets of candidate genes with common underlying biology that are dysfunctional in individuals with ASD and related to expression of a co-occurring symptoms, sleep disturbance. This work constitutes a translational bioinformatics approach beneficial to gleaning clinically useful information from WES. Given sequencing data and an initial list of candidate genes, our DBP scores can be readily calculated for any individual and relevant biological process of interest to identify clinically relevant genetic factors for a number of neurodevelopmental conditions.

## Supplementary Information

**Additional file 1: Figure S1.** Overlap in assignment of ASD/SD candidate genes with predicted damaging variants to overrepresented biological processes. Shown is the overlap among assignment of autism spectrum disorder (ASD) and/or sleep duration (SD) candidate genes with a predicted damaging variant in the SSC dataset to biological process with significant overrepresentation of genes in the pleiotropy network. The y-axis indicates the number of genes either uniquely assigned to each process, or to multiple processes, as denoted by the filled circles for processes along the x-axis. Set size indicates the number of genes assigned to any given process. Social behavior (GO:0035176), positive regulation of excitatory postsynaptic potential (GO:2000463), membrane depolarization during action potential (GO:0086010), positive regulation of synaptic transmission (GO:0051968), neuronal action potential (GO:0019228), cerebral cortex development (GO:0021987), regulation of NMDA receptor activity (GO:2000310), neuron cell-cell adhesion (GO:0007158) and regulation of ion membrane transport (GO:0034765).

**Additional file 2: Figure S2.** Correlation structure among dysfunctional biological process (DBP) scores. Shown are significant (p<0.05) Spearman’s rank correlations across DBP scores that were calculated for overrepresented processes for predicted damaging genetic variants identified in the dataset of individuals with autism spectrum disorder (ASD). Darker red indicates a stronger relationship and lighter gray indicates a weaker relationship. 

**Additional file 3: Supplementary table 1**. 

**Additional file 4: Supplementary table 2**. 

**Additional file 5: Supplementary table 3**. 

**Additional file 6: Supplementary table 4**.

## Data Availability

The source code used for the analyses is available on https://github.com/veatcho/PleiotropyGeneticRiskScores. Approved researchers can obtain the Simons Simplex Collection population dataset described in this study (https://www.sfari.org/resource/simons-simplex-collection/) by applying at https://base.sfari.org. WES data were made available via the Sequence Miner Tool 5.24.7 from WuXi NextCODE: A Contract Genomics Organization (https://www.wuxinextcode.com/). Phenotype data were downloaded directly from SFARI Base (https://base.sfari.org/).
