# Supplementary figures and images for: Calculating genetic risk for dysfunction in pleiotropic biological processes using whole exome sequencing data
Source: J Neurodev Disord. 2022 Jun 24;14:39. doi: 10.1186/s11689-022-09448-8 (PMC9233372; doi:10.1186/s11689-022-09448-8)

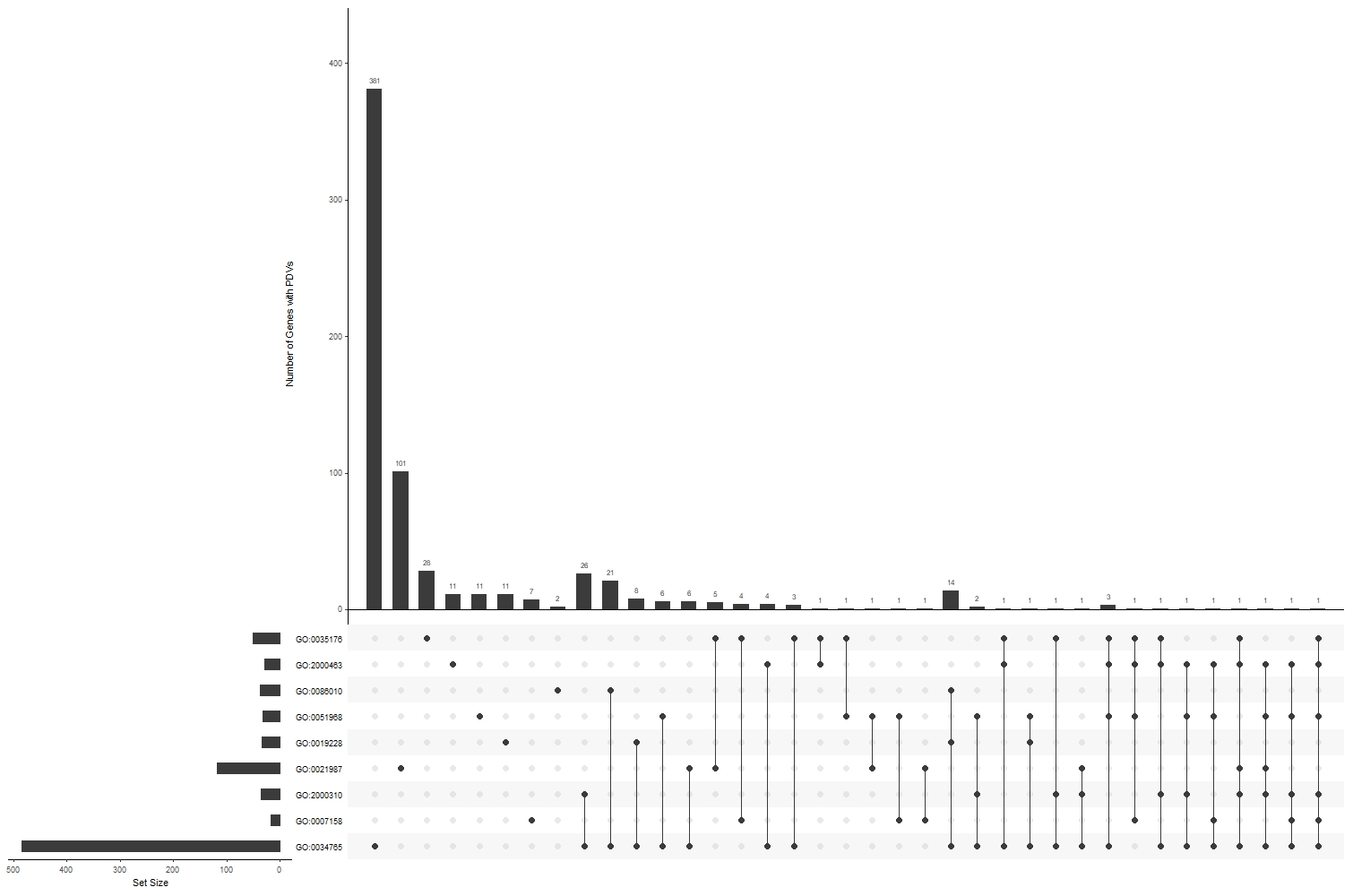

Supplement: Supplementary file 1 — Additional file 1: Figure S1. Overlap in assignment of ASD/SD candidate genes with predicted damaging variants to overrepresented biological processes. Shown is the overlap among assignment of autism spectrum disorder (ASD) and/or sleep duration (SD) candidate genes with a predicted damaging variant in the SSC dataset to biological process with significant overrepresentation of genes in the pleiotropy network. The y-axis indicates the number of genes either uniquely assigned to each process, or to multiple processes, as denoted by the filled circles for processes along the x-axis. Set size indicates the number of genes assigned to any given process. Social behavior (GO:0035176), positive regulation of excitatory postsynaptic potential (GO:2000463), membrane depolarization during action potential (GO:0086010), positive regulation of synaptic transmission (GO:0051968), neuronal action potential (GO:0019228), cerebral cortex development (GO:0021987), regulation of NMDA receptor activity (GO:2000310), neuron cell-cell adhesion (GO:0007158) and regulation of ion membrane transport (GO:0034765). [file 11689_2022_9448_MOESM1_ESM.jpeg]

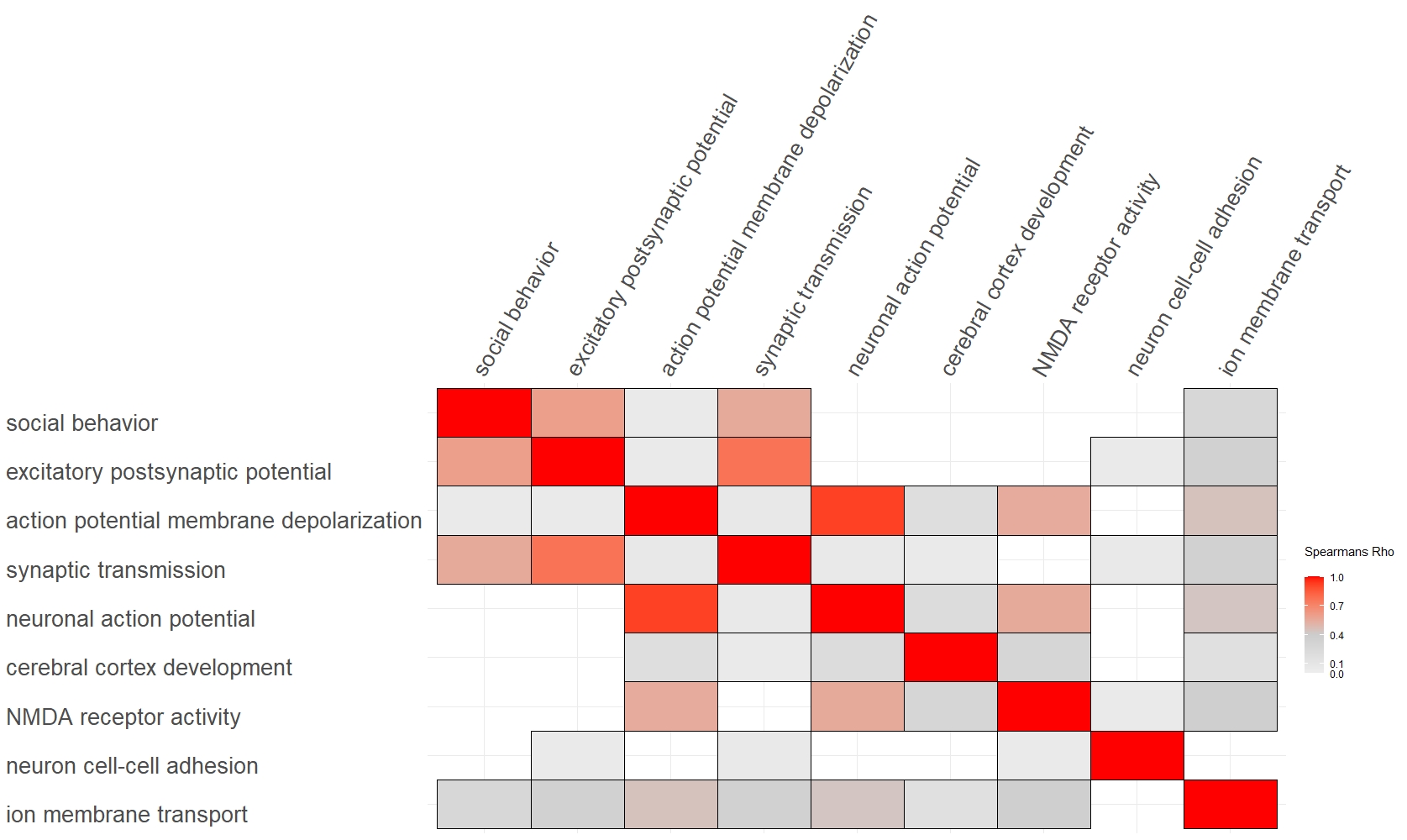

Supplement: Supplementary file 2 — Additional file 2: Figure S2. Correlation structure among dysfunctional biological process (DBP) scores. Shown are significant (p<0.05) Spearman’s rank correlations across DBP scores that were calculated for overrepresented processes for predicted damaging genetic variants identified in the dataset of individuals with autism spectrum disorder (ASD). Darker red indicates a stronger relationship and lighter gray indicates a weaker relationship. [file 11689_2022_9448_MOESM2_ESM.jpeg]
